# Priority setting in Indigenous health: assessing priority setting process and criteria that should guide the health system to improve Indigenous Australian health

**DOI:** 10.1186/1475-9276-13-45

**Published:** 2014-06-07

**Authors:** Michael E Otim, Margaret Kelaher, Ian P Anderson, Chris M Doran

**Affiliations:** 1 Poche Centre for Indigenous Health, Sydney Medical School, Edward Ford Building A27 the University of Sydney, 2006, NSW Sydney, Australia; 2 Centre for Health Programs, Policy and Economics, School of Public Health & Global Health, The University of Melbourne, 3010, VIC Melbourne, Australia; 3 Murrup Barak, Melbourne Institute for Indigenous Development, University of Melbourne, 3010, VIC Melbourne, Australia; 4 Priority Research Centre for Health Behaviour, University of Newcastle, 2308, NSW Callaghan, Australia

## Abstract

**Introduction:**

The health of Indigenous Australians is worse than that of other Australians. Most of the determinants of health are preventable and the poor health outcomes are inequitable. The Australian Government recently pledged to close that health gap. One possible way is to improve the priority setting process to ensure transparency and the use of evidence such as epidemiology, equity and economic evaluation.

The purpose of this research was to elicit the perceptions of Indigenous and non-Indigenous decision-makers on several issues related to priority setting in Indigenous-specific health care services. Specifically, we aimed to:

1. identify the criteria used to set priorities in Indigenous-specific health care services;

2. determine the level of uptake of economic evaluation evidence by decision-makers and how to improve its uptake; and

3. identify how the priority setting process can be improved from the perspective of decision-makers.

**Methods:**

We used a paper survey instrument, adapted from Mitton and colleagues’ work, and a face-to-face interview approach to elicit decision-makers’ perceptions in Indigenous-specific health care in Victoria, Australia. We used mixed methods to analyse data from the survey. Responses were summarised using descriptive statistics and content analysis. Results were reported as numbers and percentages.

**Results:**

The size of the health burden; sustainability and acceptability of interventions; historical trends/patterns; and efficiency are key criteria for making choices in Indigenous health in Victoria. There is a need for an explicit priority setting approach, which is systematic, and is able to use available data/evidence, such as economic evaluation evidence. The involvement of Indigenous Australians in the process would potentially make the process acceptable.

**Conclusions:**

An economic approach to priority setting is a potentially acceptable and useful tool for Aboriginal Community Controlled Health Services (ACCHS). It has the ability to use evidence and ensure due process at the same time. The use of evidence can ensure that health outcomes for Indigenous peoples can be maximised – hence, increase the potential for ‘closing the gap’ between Indigenous and other Australians.

## Introduction

The health status of Indigenous Australians is well below that of other Australians. The life expectancy gap is about 10.6 years, and the burden of disease is more than twice that of other Australians [1,2]. The ‘health gap’ seems to be widening over time and it is potentially avoidable, and therefore socially unjust. There are several reasons for the widening gap, such as lack of access to quality health care services, higher rates of imprisonment and poor priority setting methods in Indigenous health [3-6]. The Australian Government recently made a pledge of ‘closing the health gap’ within two generations [5]. One key issue that can be addressed in order to achieve that pledge includes improving the way government sets its priorities. For example, for a given amount of resources, how would the decision-maker choose, say, between cervical and breast cancer to fund, and how much of the selected service would be funded?

In the Australian health care system, the majority of Indigenous health care services are publicly funded. These services are provided in two ways: through the universally available health care services, referred to here as ‘mainstream services’; and through other services specifically targeting Indigenous Australians due to access issues that they face, referred to as ‘Indigenous-specific health care services’ [5,7,8]. Indigenous-specific health care services include a ‘comprehensive primary health care service’ provided by Aboriginal Community Controlled Health Services (ACCHS), drug and alcohol services, and other services provided by non-government organisations.

Mainstream services (non-primary health care, dental and other health services) are funded by the states/territories through hospitals and public health services. Other mainstream services, such as community, pharmaceutical and primary health care, are directly funded by the Australian Government through Medicare and the Pharmaceutical Benefits Scheme. Indigenous Australians are expected to utilise the universally available mainstream health care services. Due to access issues, however, they are often unable to do so. Indigenous-specific health care services are therefore meant to target Indigenous Australians as one way to address access issues.

The Australian Government mainly funds Aboriginal-specific services, and states/territories are expected to match that funding depending on the agreements entered into with the Commonwealth Government during the annual Council of Australian Governments (COAG) meetings. The delivery of the Indigenous-specific health care services is underpinned by the Framework Agreements [9,10]. These agreements are a part of the National Strategic Framework (NSF) for addressing Indigenous health issues. The framework agreements form the primary vehicle for ensuring collaboration in resource allocation, joint planning and priority setting for service delivery between key stakeholders in Indigenous health within each state and territory. The NSF is based on the principle that both Commonwealth and state levels of government are “jointly responsible for responding to the needs of all Australians (including) Indigenous and Torres Strait Islander Australians” [9,10]. It is possible that Indigenous health care funding can be diverted by governments from their original allocation; however, because the funding is normally not enough to address Aboriginal health needs, such an occurrence is not common. These services are delivered through two avenues: through the mainstream providers who are specifically targeting Indigenous Australians; and through the Indigenous community sector.

In Victoria, for example, Indigenous health funding submissions normally go through two key separate funding categories. The first is through the ‘whole of government’ funding such as housing, correctional services, employment and others. This submission goes through the head of the Ministerial Task Force on Indigenous Affairs. The second submission involves the mainstream pool of programs in which Indigenous health is treated as part of the mainstream population. When such a submission goes through the mainstream programs, it is treated as part of the departmental structure. In either case, the funding submission has to be backed by evidence. This evidence may be in the form of epidemiology data, economic evaluation evidence, or political incentives. Once the funding has been secured, the decision-makers then allocate it to specific services based on implicit reasons/criteria behind the government priority setting process. This is one of the reasons it is important to understand the government process of priority setting. Priority setting can be defined as a non-market-based process of making choices in the face of scarcity [11].

Scarcity is a fundamental economic concept, which is defined as the shortage that arises from the demand for a good or service [11,12]. From that shortage emerges the need to make choices on what to invest in and how much of the scarce resources to invest. Making such choices therefore demands that economic evaluation evidence should guide investments in Indigenous health. Economic evaluation, often referred to as ‘value for money’, can be defined as “the comparative analysis of alternative courses of action in terms of both costs and outcomes” to assess efficiency of the health care interventions [11-13], and often involves techniques such as cost effectiveness, cost benefit or cost utility [14]. From an economic perspective, decision-makers should invest scarce resources in interventions that offer ‘value for money’. Such investments can ensure that health outcomes for Indigenous Australians are maximised. It is based on that definition of priority setting that this paper takes an economic perspective when analysing priority setting.

Priority setting is multi-disciplinary and it is influenced by economics, ethics, epidemiology, political science, and other disciplines. Its approaches can be loosely grouped into: 1) implicit priority setting, where the choices and the reasons or criteria for such choices may not be clearly and publicly known; and 2) explicit priority setting, where the choices and the criteria for such choices are clearly and publicly known. However, in practice due to the complex nature of priority setting, the decision-makers may use both approaches interchangeably [15-18]. Priority setting criteria are defined as a set of values, reasons or principles on which decisions can be based. Depending on the discipline, these criteria may be referred to as preferences in economics; political drivers, incentives or public opinion in political science; or ethical principles or values when done from an ethical viewpoint [19]. For example, McDonald and Ollerenshaw have identified 13 such criteria or interconnected factors for priority setting [20]. These criteria may in turn be based on: evidence such as epidemiological data, efficiency or burden of disease estimates; solidarity; equity issues; access; or socio-economic disadvantage. Thus, criteria may be outcomes based or process based.

While the need for priority setting is widely accepted, the preferred approach is strongly contested. Carter and colleagues have noted that the key points of contention in priority setting include [21,22]: the choice between technical rigour versus due process as a preferred mechanism for undertaking priority setting; deciding whose judgement ought to inform the due process; and the choice of explicit or implicit approaches to priority setting. It is acknowledged in this paper that the preferred approach to priority setting is strongly influenced by the different levels of decision-making [23]. For example, explicit approaches may be more suitable in government decision-making levels such as Commonwealth and/or state/territory government levels, while implicit ones may be more suitable at doctor–patient levels. Currently, the government priority setting process in Indigenous health tends to be implicit. The funding often allocated does not appear to reflect the special health needs of Indigenous Australians and the reasons for such an allocation are often not known. For example, excess total expenditure per Aboriginal person compared to other Australians was 8% in 1995–96; 22% in 1998–99; 18% in 2001–02; and about 17% in 2004–05 [24-27]. It is therefore difficult for government to justify such marginal increases in funding given the health gap. This paper focusses on explicit priority setting as a macro and meso approach for the government policy of closing the gap to be achieved in a transparent manner and ensuring accountability.

An assessment of explicit priority setting attempted in several countries such as the United Kingdom, New Zealand and Sweden by Sabik and Lie yielded mixed results [28]. They found that it is important to base priority setting on criteria that are of importance to the society affected by the exercise, such as equality, equity, human dignity, human rights and autonomy [16,18,29,30]. This assessment demonstrates that, although explicit priority setting is not necessarily and universally more valid and more reliable than implicit judgement, it may be preferred if it allows Indigenous Australians to participate and express their preferences. It therefore has potential to enhance transparency.

The key challenge however, is how explicit priority setting should be implemented in Australian Indigenous health. Shiell and Mooney argue that establishing a statement of the broad principles or criteria that should guide the health system and the trade-offs that the citizens are prepared to make between the stated principles in Indigenous health could be the starting point [23]. This would be followed by employing a mechanism that maximises health for the given resources, and a framework for providing management incentives and infrastructure.

The purpose of this research was to elicit the perceptions of Indigenous and non-Indigenous decision-makers on several issues related to priority setting in Indigenous-specific health care services. Specifically the objectives of this study were to:

1. identify the criteria being used to set priorities in Indigenous-specific health care services;

2. determine the level of uptake of economic evaluation evidence by decision-makers and how to improve its uptake; and

3. identify how the priority setting process can be improved from the perspective of decision-makers.

## Methods

We undertook a descriptive cross-sectional survey of the key decision-makers involved in Indigenous-specific health care services to elicit their perceptions, views and values on a range of issues associated with priority setting in Victoria.

A ‘snowball’ process was used to identify all participants and their extended associations, through previous acquaintances, involved in resource allocation and/or priority setting in Indigenous-specific health care services [31,32]. A questionnaire was researcher-administered through a face-to-face meeting and analysis involved both descriptive statistics and content analysis. Ethics approval was obtained from the University of Melbourne and support was obtained from the Indigenous community-controlled sector in Victoria.

### The sample

Participants of this study involved two groups of decision-makers in the state of Victoria: staff from the then Department of Human Services (DHS) a state-level government funding agency; and Chief Executive Officers (CEOs) and/or their representatives from various ACCHS, the providers of comprehensive primary health care services to Indigenous community members.

Twenty-four participants from the DHS participated in the survey and we made every attempt to identify all decision-makers involved in Aboriginal-specific health care services throughout the state. Almost all the regions were represented. The DHS sample is a government agency which is primarily involved in the funding of the services for both Indigenous and non-Indigenous Australians in Victoria. They are not involved in the delivery of Indigenous-specific health care services.

We surveyed 26 respondents from the ACCHS, who are mainly providers of comprehensive primary health care services to Indigenous peoples in their communities. All ACCHS in Victoria were contacted and requested to participate in the survey. The key decision-maker in ACCHS is the CEO with the help of his/her deputies. However, the ACCHS Board have to approve their plans. The ACCHS are funded by the Department of Human Services (DHS), the Commonwealth Office of Aboriginal and Torres Strait Islander Health (OATSIH) and from non-government organisations (NGO). Thus, ACCHS often have to deliver services according to the criteria prescribed by the funder such as the DHS. Where the ACCHS have freedom to choose how to spend the funding, ACCHS criteria may be different from those of the DHS.

By choosing the DHS and ACCHS, we aimed to identify whether there was consistency in the criteria/principles that guide priority setting based on the perspectives of the funder and provider. Given that both groups of respondents aim to achieve the same objective – improve the health outcomes of Indigenous Australians in Victoria – can improvement in resource allocation and priority setting necessarily lead to improvement in health outcomes?

Other groups such as hospitals, general practitioners (GPs), community pharmacists, community health services and Commonwealth workers were excluded because most of them are not involved in the delivery of a significant amount of Indigenous-specific services.

Overall, 67% (26/39) of the DHS respondents were Directors and the rest were senior executives – heads of units. As for those from the ACCHS sector, 65% (24/37) of the ACCHS staff who agreed to participate were mainly CEOs and the rest were senior executives, deputy CEOs and managers of the health services. None of the DHS respondents identified themselves as Indigenous people, compared to 58% of ACCHS respondents. Of the DHS respondents, 46% had served for more than two years in their respective positions, compared to nearly 50% of ACCHS respondents.

### Recruitment

To identify participants for this study, a ‘snowball’ sampling process was used until all the participants involved in priority setting in Aboriginal-specific health care service from the organisations were identified. Snowballing is a tool for selecting participants from extended associations, through previous acquaintances, to find people involved in Indigenous-specific health care services [31,32]. The key reason for choosing snowball sampling involved its ability to allow us to identify other members within the DHS and ACCHS involved in Indigenous-specific health care services who could be interviewed.

In the DHS, we started by approaching the Director of Public Health who had a working relationship with us. He in turn identified other potential respondents. For ACCHS respondents, we contacted the CEO of the Victorian Aboriginal Community Controlled Health Organisation (VACCHO) for permission to contact its member organisations. This ensured that potential participants felt encouraged to participate in the study. Letters were then sent out via email inviting them to participate and this was followed by a phone call around a week later to arrange a face-to-face meeting. After the face-to-face meeting, they were asked to identify potential participants, whom we then approached. This process was continued until we had exhausted all potential participants.

### Data collection instrument

We used a paper-based survey instrument, which was adapted from the previous work done by Mitton and colleagues [17,33-36] for both ACCHS and DHS participants. The original questionnaire had both open-ended and closed-ended questions that related to the criteria used to set priorities, the sources of data used and how the priority setting process can be improved. The closed-ended questions each had a list of all possible answers, including Aboriginal specific criteria that were generated from a review of the literature, from which the participants could choose. We also added an open-ended option termed “other” at the end of each question where participants could report any other answers that were not listed for that question. Answers for this section varied from one word to a couple of brief sentences depending on the question.

The questions and modified answers were subsequently piloted with staff and students in the School of Population Health, The University of Melbourne. Based on the pilot study, we simplified some of the questions.

The second modification involved face-to-face delivery of the questionnaire. Initially, we intended to use an email questionnaire, but the pilot study indicated that, although simplified, some questions were still technical and may have required explanations and so face-to-face administration of the questionnaire was chosen. In addition, a list of definitions was provided to the respondents together with the questionnaire, prior to the face-to-face meeting.

Respondents were required to tick the answers in the questionnaire that they used as a basis for setting priorities, and add any other concepts or terms that where missing from the list. If a respondent had mentioned some terms that were not amongst the ones we had identified from the literature, we asked the respondents to explain what they meant when they used that term.

We interviewed respondents between December 2007 and March 2008. The questionnaire was sent out in advance to the participants. Following this, face-to-face meetings were arranged with each participant to administer the questionnaire. This allowed clarification of the key concepts by the researcher during the administration of the questionnaire and enhanced accurate elicitation of the views [4]. This also allowed the researcher to identify early on in the meeting whether the participant was sufficiently involved in Indigenous-specific priority setting. In the rare case that the participants were not involved, the interview was terminated. Respondents were required to tick the relevant responses from the list of the possible responses. Where a response was not listed, respondents were required to write it down under ‘other’ and/or elaborate verbally. The glossary of the key terms used in the survey was included in the plain language statement that accompanied the interview questions. At the time of the interview, respondents were reminded to ask for explanations in cases where the questions were unclear. We sought participants’ consent for the interview to be recorded in order to ensure accuracy of the responses to the open-ended questions. The interview transcripts were later mailed to the respondents for confirmation.

Personal data of decision-makers were collected, such as their Aboriginality, role in their respective organisations and length of service, involvement in priority setting and/or resource allocation, and any exposure to health economics. Other data involved current priority setting process, assessment of the process and how the process could be improved (Table 1) [33].

**Table 1:** Structure of the questionnaire and definition of terms

	**Questions:**
	**Background information**
	• Are you of Aboriginal or Torres Strait Islander descent?
	• Are you involved in Indigenous-specific priority setting within your organisation?
**Part I A**	**Current process of setting priorities and allocation of resources**
	• What is considered to be the key objectives of this organisation?
	• What sources of information are used in determining short-term and long-term priorities?
	• Once priorities are defined, how are decisions made to allocate resources across organisations/programs?
	• How is the benefit from programs/interventions viewed in your organisation?
	• On what basis are decisions made in resource allocation and service/program activity?
**Part I B**	**Assessment of the current priority setting process**
	• In your opinion, how does the process of priority setting perform?
	• What are the specific strengths of the current process?
	• What are the challenges of the current approach?
**Part I**	**Improving the process of priority setting**
	• How could the current process of setting priorities be improved?
	• Do you think there is enough transparency/explicitness built in it?
	• Do you think the use of economic principles and/or evidence from economic evaluation could improve the process of priority setting?
	**Definition of terms:**
Cultural Security	Involves a commitment that the construct and provision of services offered by the health system will not compromise the legitimate cultural rights, views, values and expectations of Aboriginal people. It is the recognition, appreciation of, and the response to the impact of cultural diversity on the utilisation and provision of effective services to Aboriginal people.
Effectiveness	The extent to which a service ‘works’ and achieves its objectives in real life.
Efficiency	A relationship between resource use and outcomes. When a service is deemed ‘inefficient’, it implies that greater outcomes could be achieved with the same resources or that the same outcomes could be achieved with fewer resources.
Equity	Equity relates to fairness or social justice. It involves ethical judgements about the fairness of the distribution of health outcomes, accessibility of health services or exposure to health-threatening hazards.
Explicit approach	This is a priority setting approach in which both the decisions and the basis for the decisions by a decision-maker (i.e. data sets, findings, criteria of judgement, etc.) are clearly known and stated.
Implicit approach	This is a priority setting approach where the decisions made by a decision-maker and the reasons for those choices are not clearly stated.
Key decision-makers	Senior management (such as senior project officers) involved in setting priorities or making decisions in resource allocation.
Marginal analysis:	Marginal analysis is an economic technique that looks at the costs and outcomes of potential changes to the current mix of services provided. It focuses on incremental changes to resources, programs, cost or outcomes of a service. For example, what happens if a little less or more of an activity takes place.
Priority Setting:	Describes the process of determining what comes first on the list (in the order of importance) of activities/programs of an organisation with a given budget.
Resource allocation	Describes the process of determining how funding is distributed or divided up across programs or services.
Stakeholder involvement	The degree to which stakeholders are involved in the process of setting priorities. This may be none, a little or a lot.
Lack of resources	Where there are inadequate resources, such as funds, available to fulfil organisational processes and deliver services.
Indigenous community as a data source:	The data used for priority setting is sourced from the views or preferences of the Indigenous community, mainly as a formal process through studies or community consultations or public opinion.

### Data analysis

Closed-ended and open-ended responses were analysed using content analysis, “a research technique for the objective, systematic, and quantitative description of manifest content of communications” [37-39]. For analysis, we entered the responses into an Excel spreadsheet and manually counted the number of times each concept was mentioned. The more times a concept was mentioned, the more likely it is to be used by decision-makers as criteria. Where answers were reported for the open-ended option, we re-allocate those terms to one of the a priori criteria or created new concepts with definitions. Where there was uncertainty of the relevance of open-ended answers, we looked through the literature to see if such criteria were potentially important for guiding priority setting. These results were expressed as descriptive statistics and supplemented with explanatory quotes that captured or highlighted key issues that emerged from the survey.

Whilst there may have been difference in the weights participants attached to their responses, particular where there were multiple answers to a question, this was not undertaken. Instead, equal weighting was given to all the responses from each interviewee. This aligned with the aim of the study, which was to identify criteria used for decision making in Indigenous health, and not to necessarily give weight to specific criteria. For each sample, the frequency each criteria was cited was determined to reflect the level of use in priority setting by that group.

The focus of the study was also on examining the similarities and differences in responses between decision makers on whether they were a ‘funder’ or ‘provider’. Therefore, whilst the position or rank of each participant within their organisation could have influenced the participant’s responses, this was not taken into account as weighting answers was not done. Instead the aggregated responses were examined as a means of comparing the different criteria used by funders and providers and their assessment of the process of priority setting overall.

Results of the closed-ended questions were reported using descriptive statistics and open ones were also reported using descriptive statistics but augmented by quotations where possible and appropriate. The final score for each sample involved the number of times (n) or proportion (%) of participants who gave a particular response. The criteria that the majority of the decision-makers cited were considered the likely bases for decision-making.

## Results

### Key objectives that organisations pursue

Results from this study indicate that the perspective taken by the DHS staff when setting priorities is that of the whole population, requiring equity considerations, whereas the ACCHS focus mainly on the Indigenous population. For these reasons, the objectives (Table 2), criteria and needs of the two samples may differ. Indeed, the DHS respondents identified effectiveness and equity as the most important objectives that they pursue in Indigenous-specific health care services. ACCHS respondents on the other hand identified impact of the intervention on the health of the Indigenous Australians and sustainability as key objectives, with effectiveness and equity being low among objectives in their community organisations.

**Table 2:** Key objectives that organisations pursue

**Objectives**	**Responses**			
	**DHS (n) %**	**Rank**	**DHS (n) %**	**Rank**
Impact on health	(11) 42%	7^th^	(19) 79%	1^st^
Sustainability	(18) 69%	3^rd^	(18) 75%	2^nd^
Access to services	(13) 50%	5^th^	(16) 67%	3^rd^
Acceptability	(15) 58%	4^th^	(15) 63%	4^th^
Effectiveness	(20) 77%	1st	(14) 58%	5^th^
Efficiency	(11) 42%	6^th^	(12) 50%	6^th^
Feasibility	(8) 31%	8^th^	(9) 38%	7^th^
Affordability	(7) 27%	9^th^	(9) 38%	8^th^
Equity	(18) 69%	2^nd^	(7) 29%	9^th^

### Perceptions of the current process of setting priorities

With respect to the sources of data currently used in decision-making (Table 3), ACCHS respondents indicated that a key source of both short and long-term data is the Indigenous community (short-term: 71%; long-term: 67%) and needs assessment (short-term: 54%; long-term: 54%). For the DHS respondents, results were very similar for both short- and long-term sources: state/federal policy documents (short-term: 58%; long-term: 73%) and evidence (short-term: 62%; long-term: 73%).

**Table 3:** Data sources and criteria for priority setting

**Data sources used by DHS and ACCHS for priority setting**					
**Short term sources of data**	**DHS (n) %**	**ACCHS (n) %**	**Long term sources of data**	**DHS (n) %**	**ACCHS (n) %**
Evidence: e.g. economic evaluation, epidemiology	(16) 62%	(9) 38%	State/Federal policy documents	(19) 73%	(12) 50%
State/Federal policy documents	(15) 58%	(9) 38%	Evidence: e.g. economic evaluation, epidemiology	(19) 73%	(6) 25%
Indigenous community (Indigenous views/preferences)	(13) 50%	(17) 71%	Key organisation objectives	(17) 65%	(10) 42%
Key organisation objectives	(12) 46%	(12) 50%	Indigenous community	(14) 54%	(16) 67%
Funds available/financial directive	(10) 38%	(12) 50%	Needs assessment	(10) 38%	(13) 54%
Needs assessment	(8) 31%	(13) 54%	Funds available/financial directive	(8) 31%	(11) 46%
Mainstream public opinion	(7) 27%	(12) 50%	Three-year business plan	(6) 23%	(11) 46%
**Criteria used by DHS and ACCHS decision-makers for priority setting**					
**Across programs**	**DHS (n) %**	**ACCHS (n) %**	**Within programs**	**DHS (n) %**	**ACCHS (n) %**
Size of the health problem	(18) 69%	(15) 63%	Feasibility/sustainability	(14) 54%	(12) 50%
Feasibility/sustainability	(13) 50%	(10) 42%	Size of the health problem	(13) 50%	(17) 71%
Equity	(11) 42%	(8) 10%	Equity	(12) 46%	(4) 17%
Political ‘hot spots’	(11) 42%	(4) 17%	Acceptability	(10) 38%	(13) 54%
Acceptability	(10) 38%	(13) 54%	Efficiency	(7) 27%	(10) 42%
Access to services	(10) 38%	(8) 33%	Access to services	(6) 23%	(10) 42%
Historical trends/patterns	(9) 35%	(10) 42%	Historical trends/patterns	(5) 19%	(12) 50%
Efficiency	(3) 12%	(11) 46%	Political ‘hot spots’	(4) 15%	(2) 8%

The DHS respondents identified the following as key criteria for priority setting across and within programs. These included: size of the burden; feasibility/sustainability; equity; and acceptability as important criteria (Table 3). For example, one DHS respondent indicated that:

“… for me the most important issues would be the equity … and the size of the health burden would be an issue but not the overriding factor.”

The ACCHS on the other hand, revealed that the size of the health burden and the acceptability of the priority setting process were the key criteria for priority setting both across and within the programs (Table 3). The importance of feasibility/sustainability could be seen to be consistent with the need for programs/services that can be implemented and can continue to be delivered to the Indigenous Australians.

Further, for the ACCHS, equity is not an issue since the communities they may be responsible for could be considered fairly homogenous. They considered efficiency and impact of the services on the community members as priority issues. They were also concerned with mechanisms of making the funds go the furthest, since the communities under their jurisdictions have multiple needs, which can be interpreted as the need for economic evaluation evidence and need for mechanisms that ensure that resources can be moved easily across and within programs.

On the question of what constitutes benefit from interventions, we asked respondents to indicate outcomes they would like to see delivered by health care services to Indigenous Australians. Respondents from both samples indicated that benefits should not only include individual health gain (majority of the members revealed), but other benefits such as community health gain, equity and cultural security. Cultural security is defined as “the delivery of health services is of such a quality that no one person is afforded a less favourable outcome simply because they hold a different cultural outlook” [40]. The Indigenous community, as a data source, means that priority setting data is sourced from the Indigenous community, mainly as a formal process through studies, community consultations or public opinion.

### Assessment of the current priority setting process

About 73% of the DHS respondents reported that the resource allocation process did not work well. As for the ACCHS respondents, the majority (58%) reported that the priority setting process worked well. ACCHS respondents were particularly interested in the criteria that the funding agencies use for allocating resources. One respondent said:

“I would be happy to know what reasons the funders use to select programs/services to fund. Most importantly, I would like to know the reasons why they have not funded some of our activities.”

Respondents identified several key challenges that they face when they undertake priority setting. The DHS respondents reported that the lack of good quality data (58%) and lack of expertise (54%) were the key issues they were faced with (see Table 4). These issues would have an impact on the role of economic evidence in priority setting in the DHS. The ACCHS respondents reported lack of resources and the tendency for the process to be crisis oriented as the key challenges (Table 4). These two issues were quite different from those faced by the DHS respondents.

**Table 4:** Challenges of priority setting process in Indigenous health

**Challenges:**	**DHS (n) %**	**ACCHS (n) %**
Lack of good quality data	(15) 58%	(10) 42%
Lack of expertise to undertake priority setting	(14) 54%	(12) 50%
Crisis oriented planning	(7) 27%	(15) 63%
Lack of resources	(7) 27%	(19) 79%
Not systematic	(6) 23%	(6) 25%
No/little public or stakeholder involvement	(5) 19%	(8) 14%
Implicit decision-making	(5) 19%	(4) 17%
Lack of time	(5) 19%	(6) 25%

### Improving the priority setting process

To improve priority setting, 81% of the DHS respondents indicated that better data and strength of evidence was needed. They also indicated that having a longer-term view (54%) when the resources are being allocated would improve the priority setting process (Table 5). Others identified the need to have a mechanism that can be used to systematically shift resources across programs (35%), while being as transparent as possible, with less political influence, to ensure explicit priority setting. ACCHS respondents ranked better data/strength of evidence (63%) and longer-term view (63%) as the key issues to be addressed in order to improve priority setting.

**Table 5:** Improving the priority setting process

**Improving the process**	**DHS (n) %**	**ACCHS (n) %**	**Uptake of economic evaluation**	**DHS (n) %**	**ACCHS (n) %**
Better data and evidence	(21) 81%	(15) 63%	Up-skilling of staff	(15) 58%	(12) 50
Longer term view	(14) 54%	(15) 63%	Better data from studies	(14) 54%	(10) 42%
Mechanism for shifting resources across programs required	(9) 35%	(4) 17%	Longer term view taken	(10) 38%	(9) 38%
Systematic process of priority setting	(8) 31%	(9) 38%	Better communication strategy	(10) 38%	(8) 33%
More explicit/transparent methods	(7) 27%	(8) 33%	More education on the credibility of economic evaluation	(10) 38%	(7) 29%
Examining the margin when choosing to reduce or increase resources or activity	(2) 8%	1 (4%)	Timely data from decision-makers	(10) 38%	(7) 29%
Less political influence	(2) 8%	(3) 13%	Transparent economic models	(9) 35%	(6) 25%

With regards to availability of in-house data for priority setting, one respondent from an ACCHS argued that the funding agencies ought to recognise that ACCHS have the data on their records. One respondent remarked:

“It is just a matter of someone with expertise coming to comb through our records or collect the data through our organisation, rather than borrowing inappropriate data from elsewhere to use for priority setting.”

The above information vindicates the need for an explicit priority setting process that makes use of better evidence and data, such as from economic evaluations. In particular, the need to up-skill staff in health economics and/or more training of health economists (50%) featured significantly from ACCHS respondents as a means to increase the use of economic evaluation evidence. The DHS respondents also indicated that up-skilling of staff in health economics and/or more training of health economists (58%) would improve the uptake of economic evaluation evidence. Having a longer-term view, together with better communication strategy and more education on the credibility of economic evaluation, would improve the uptake of economic evidence. One respondent said:

“The trouble with academics is that they take too long to get results to us. When you ask them for some information, they tell you that results for an economic evaluation will take two years to be produced. By then priorities will have changed.”

## Discussion

The primary purpose of this study was to identify, codify and better understand the current process of setting priorities and allocating resources in Indigenous-specific health care services. In particular, identifying the criteria used by decision-makers, assessing and improving the priority setting process, particularly the uptake of economic evaluation evidence in priority setting, were the key objectives of this study. We interviewed key decision-makers in Indigenous-specific health from both the government and Indigenous community sector.

McDonald and Ollerenshaw identified about 13 interconnected factors or criteria for priority setting [20], which are consistent with our results, as we acknowledge the role played by other disciplines or criteria when a decision-maker is faced with the task of making choices, either in a hurry or in the long run. Our study revealed that criteria identified by participants were similar for the two organisations; however, there were differences around the place for equity. The differences are likely to stem from the different primary roles that the two organisations are engaged in. As already indicated, the DHS is generally a funding agency and may therefore consider equity and other criteria differently when allocating resources for the whole Victorian population. ACCHS on the other hand, being primarily service providers for the Indigenous population, may not have their focus on equity and similar criteria since they tend to look after a defined population. Further, we did not elicit the intensity of the preferences of the respondents for the criteria identified, but scientific evidence emerged as the criterion that most respondents listed. These results are consistent with results from Monk et al. [41]; however, our results may not be useful for making trade-offs between criteria when choices are being made.

The findings from this study also build on work by Mitton and colleagues identifying criteria that have been used in Canada, Uganda and other parts of the world. They have all identified similar criteria, albeit with some variations in intensities. Social justice principles were also identified by ACCHS respondents in our study and these results were consistent with the above studies. To establish the intensities of these criteria would be the next step of research.

One other key issue in priority setting involves the choice between explicit and implicit priority setting. Results from the survey indicate that respondents favoured the key steps that decision-makers should follow when undertaking explicit priority setting, as identified by Shiell and Mooney [23]. Some of these steps involve establishing an Aboriginal health constitution; identify decision-making criteria and their trade-offs that the community is prepared to make; the use of evidence to support decision-making; and marginal analysis. Our study identified some of the criteria and principles that should appear in an Aboriginal health constitution and how to aggregate them into organisation processes.

This survey also indicated that one of the key sources of data that should be used for priority setting is the Indigenous community. This finding is consistent with the model used by Fredericks and colleagues to engage women in a meaningful manner in setting priorities for the Women’s Health Strategy [42]. Further, this finding contributes to one of the key issues that often emerges in the priority setting debate as to whose views should inform priority setting – should it be Indigenous people or decision makers [41] It was reported that having the involvement of Indigenous people in the exercise itself made the participants support the results and was likely to enhance acceptability of the results [20].

On assessing the process of decision-making in Indigenous health, most respondents revealed that it is not performing as well as they would like it to be. Four key issues were identified. First, when priorities are being set, there is need for a systematic and explicit transparent process. These results confirmed one of the steps emphasised by Shiell and Mooney [23].

Second, there is need for a timely and better “strength of evidence” base, especially in the form of economic evaluation results. However, the uptake of economic evaluation evidence required the up-skilling of staff in health economics and/or more training of health economists. Further, not many decision-makers suggested an area where health economics is a priority.

Third, the majority of the respondents from both the DHS and ACCHS indicated that they needed a mechanism for shifting resources (a decision rule) within programs, which aids decision-making at the margin, and not just the use of the concept of total need. For example, once the DHS funding allocations have been approved, each portfolio or unit funds its own activities in Indigenous health, often in a prescriptive manner. Making decisions across programs rarely happens and resource shifts between programs are very restricted, except in a few areas such as funding from the ‘Home and Community Access’ program and the Medicare funding for GP services. These are the main areas where the organisations have liberty to move resources across programs, implying that certain priority setting criteria may be applicable to the DHS but not to ACCHS. This reflected the poor uptake of economic evaluation evidence and the need for an economic approach for priority setting.

Indeed one of the key contributions of this paper, given the Framework Agreements principles of joint priority setting is comparing priority setting processes (and links) between two different organisations which work together to achieve the same aim – improving the health of Indigenous Australians given the limited resources. The linkage is in ensuring that the health service provider is guided by the same criteria that guide the funding agency. The funder ought to focus on outcomes when allocating resources and the service provider should also focus on outcomes when setting priorities [23]. This has been explained more in the context of the specific roles of the two samples are and where they meet.

Fourth, depending on the nature of the problem being addressed, the views of the community might be better taken into consideration rather than relying on only those of the bureaucracy. Involving Indigenous representatives in the priority setting process would enhance acceptability, support and adoption of the results. This was also demonstrated in our study and similar results were identified when women participated in a priority setting exercise for the Aboriginal and Torres Strait Islander health strategy [42].

### Limitations of this study

Our approach to eliciting the perceptions of the decision-makers involved the use of a hard copy questionnaire and it has been used extensively around the world. Our approach is adapted from the work done by Mitton and colleagues in Canada, Australia and the UK [34,43-47], to identify participants using the snowball process. If we had used a collaborative approach [48] in developing priorities, we could probably have developed a different set of criteria. We do recognise the importance of a collaborative approach [42] and other approaches such as contingent valuation.

We were aware of the fundamental issue with surveys, which involves the presumption that a specific moral theory underlies the interview questions, and that the respondents are bound by that theory [49,50]. However, respondents may be tempted to ignore social values in their responses and instead choose to concentrate on their personal interests by focusing on the official government line. To overcome this, respondents were continually reminded during the face-to-face meeting that their responses would be kept confidential. Further, the respondents were decision-makers who are involved in priority setting in Indigenous-specific health care services. Had we included other decision-makers involved in other health care services utilised by Indigenous peoples, the results could have been different. For that reason, these results should be used with caution.

## Conclusion

We conclude that in order for the health of Indigenous Australians to be improved, decisions made by both the funders and the providers should be explicit and take into account what the returns from investments entail. For priority setting process in Indigenous health to improve, funding agencies need to align their funding criteria with the priority setting criteria of the providers. Specifically, both funding agencies and providers should focus on outcomes and make use of economic evaluation evidence when undertaking priority setting processes. This would allow the decision-making process to be systematic, transparent, and to take a longer-term view. Furthermore, there is a need for involvement of the Indigenous representatives to enhance acceptability, support and adoption of the results.

The results from this study are likely to inform the debate on the appropriateness of the existing criteria for priority setting, not only in Australia but other countries, given the scarcity of the resources. This will likely strengthen the need for economic evaluation evidence and will also generate discussion on the relative importance of such evidence. Further, by identifying ways to elicit Indigenous peoples’ views in the priority setting process, these results will emphasise and further explain the role of Indigenous values in determining priorities and choices for closing the health gap. It will also highlight the need to take into account the views of both Indigenous and non-Indigenous Australians All these factors will potentially ensure that the choices made by decision-makers will maximise the health of Indigenous peoples and contribute to the closing of the health gap between Indigenous and other Australians.

## Competing interest

The authors declare that they have no competing interests.

## Authors’ contributions

MEO: Developing and initiating the idea for writing manuscript, Data collecting, cleaning and analysis, drafting manuscript. MK, IPA and CMD: Advising on methodology and direction of constructing manuscript. Correcting the manuscript. All authors read and approved the final manuscript.
